# Studying Secondary Growth and Bast Fiber Development: The Hemp Hypocotyl Peeks behind the Wall

**DOI:** 10.3389/fpls.2016.01733

**Published:** 2016-11-18

**Authors:** Marc Behr, Sylvain Legay, Eva Žižková, Václav Motyka, Petre I. Dobrev, Jean-Francois Hausman, Stanley Lutts, Gea Guerriero

**Affiliations:** ^1^Environmental Research and Innovation Department, Luxembourg Institute of Science and TechnologyEsch-sur-Alzette, Luxembourg; ^2^Groupe de Recherche en Physiologie Végétale, Earth and Life Institute-Agronomy, Université catholique de LouvainLouvain-la-Neuve, Belgium; ^3^Institute of Experimental Botany, The Czech Academy of SciencesPrague, Czechia

**Keywords:** *Cannabis sativa*, hypocotyl, bast fibers, cell wall, RNA-Seq, immunohistochemistry, phytohormones

## Abstract

*Cannabis sativa* L. is an annual herbaceous crop grown for the production of long extraxylary fibers, the bast fibers, rich in cellulose and used both in the textile and biocomposite sectors. Despite being herbaceous, hemp undergoes secondary growth and this is well exemplified by the hypocotyl. The hypocotyl was already shown to be a suitable model to study secondary growth in other herbaceous species, namely *Arabidopsis thaliana* and it shows an important practical advantage, i.e., elongation and radial thickening are temporally separated. This study focuses on the mechanisms marking the transition from primary to secondary growth in the hemp hypocotyl by analysing the suite of events accompanying vascular tissue and bast fiber development. Transcriptomics, imaging and quantification of phytohormones were carried out on four representative developmental stages (i.e., 6–9–15–20 days after sowing) to provide a comprehensive overview of the events associated with primary and secondary growth in hemp. This multidisciplinary approach provides cell wall-related snapshots of the growing hemp hypocotyl and identifies marker genes associated with the young (expansins, β-galactosidases, and transcription factors involved in light-related processes) and the older hypocotyl (secondary cell wall biosynthetic genes and transcription factors).

## Introduction

Hemp (*Cannabis sativa* L.) is a herbaceous plant native to Central Asia known for its medicinal and textile applications for over 10,000 years. There is currently a renewed interest in hemp, because of its multitude of applications, notably in the construction and biocomposite sectors (Andre et al., 2016; Guerriero et al., 2016). As an example, the woody fibers of industrial hemp are used to manufacture a concrete-like material known as “hempcrete,” which is very light in weight, while the cellulosic fibers find application in the biocomposite sector for the creation of bioplastics.

The stem of *C. sativa* provides two types of fibers, i.e., the xylem and the phloem fibers. The latter are known as bast fibers. The cell walls of xylem fibers are impregnated with lignin to ensure strength and resistance to negative sap pressure (Wang et al., 2013), while those of the bast fibers are mainly made of crystalline cellulose (and can account for up to 75–80% of the dry mass; Guerriero et al., 2013). The other components of bast fiber cell walls are hemicelluloses (4%, including xyloglucan in the PCW and xylan in the SCW), pectins (4%), proteins (3%), lignin (2%) and traces of phenolic acids (<0.01%) (Crônier et al., 2005).

The cell wall is a highly dynamic and complex compartment, because its composition undergoes massive modifications as the plant develops (Cosgrove, 2005; Zhang et al., 2014). A typical example is the transition from primary to secondary growth in plants, a process requiring the shift from a phase characterized by active elongation to a stage of stem thickening (van Raemdonck et al., 2005; Zhong and Ye, 2007). Secondary growth is a relevant process, as it generates wood, an important renewable resource for humankind (Guerriero et al., 2014). Following the primary elongation phase, it is responsible for the radial growth of the stem and the root. Secondary growth is a complex phenomenon to study from a molecular perspective, as it is tuned by both exogenous factors (Moura et al., 2010) and phytohormones (via transcriptional master regulators; Didi et al., 2015). The diversity of cell wall composition has inspired the molecular study of various models for secondary growth, from trees (Tocquard et al., 2014) to specific tissues of herbaceous plants (Zhong et al., 2008; Taylor-Teeples et al., 2015).

In trees, the annual girth increase is the result of the activity of a secondary meristem, the vascular cambium. Both the vascular cambium and the genes involved in secondary growth are not restricted to trees, but are present in herbaceous species such as the small weed *Arabidopsis thaliana*. Secondary growth has been extensively studied in both the hypocotyl and root of this model organism (Sibout et al., 2008). In the *A. thaliana* hypocotyl the phases of elongation and girth increase are temporally uncoupled (Ragni et al., 2011), which enables experimenters to focus on different cell wall-related aspects of the same structure.

Among herbaceous plants, fiber crops also present secondary growth. In particular, the hypocotyl of flax, *Linum usitatissimum* L., was shown to be a valid system to study the molecular processes occurring during the stages of elongation and subsequent thickening of bast fibers (Roach and Deyholos, 2008). The authors showed indeed that bast fiber development in adult flax stems occurs following the same two stages of elongation and secondary wall deposition arising in the young hypocotyls. These two systems are therefore complementary for the study of bast fiber development. In the adult stem of fiber crops, a lignification gradient is observed: the basal internodes of the stem are more lignified than the younger elongating ones. The transition between the rapidly elongating and the lignified internodes of the stem occurs at a characteristic spot in flax, the so-called “snap point” (Gorshkova et al., 2003). The snap point therefore physically marks a zone below which the mechanical properties of bast fibers change considerably (Gorshkova et al., 2003). Hemp stems show the same basipetal gradient of lignification, with older regions of the stems characterized by a well-developed xylem and both primary and secondary bast fibers (Crônier et al., 2005). Notably, the developing hemp hypocotyl shows the same tissue organization, i.e., at younger stages the cells are elongating, while at older ones the tissues are more lignified, with the appearance of secondary bast fibers. As these cells originate from the cambium rather than the pro-cambium as in flax (Snegireva et al., 2015), the hemp hypocotyl is suitable to study their biosynthesis from a molecular point of view.

With the aim of proposing an alternative simple system to study secondary growth, the suitability of the hemp hypocotyl is here validated. In line with the study of Roach and Deyholos (2008), four sequential time points (6–9–15–20 days after sowing) are here investigated using a cross-disciplinary approach: transcriptomics (RNA-Seq) and the quantification of phytohormones have been coupled to optical and confocal microscopy observations. The goal is to decipher the molecular events involved in the radial growth and bast fiber development of the hemp hypocotyl. The results here shown contribute to add fundamental knowledge to the study of secondary growth and fiber formation in an economically important plant.

## Materials and Methods

### Plant Material and RNA Extraction

*Cannabis sativa* (cv. Santhica 27) hypocotyls of 6, 9, 15, and 20 days after sowing were grown in controlled conditions in incubators following a cycle of 16 h light 25°C/8 h dark 20°C. Three biological replicates per time point were analyzed. Each biological replicate consisted of 20 hypocotyls randomly selected among all incubators. The pooling of hypocotyls was needed because of the quantity of material required for transcriptomics. In order to bring to a minimum this pooling bias, a high number of hypocotyls were pooled together. By doing so, the power to detect differentially expressed genes within the four populations increased (Rajkumar et al., 2015). Sampling was performed on a single experimental batch to minimize technical variability. Samples were immediately frozen in liquid nitrogen and conserved at -80°C until RNA extraction. The sampled hypocotyls were crushed to a fine powder using a mortar, a pestle and liquid nitrogen. Total RNA was extracted using the RNeasy Plant Mini Kit (Qiagen), treated with DNase I on column, quantified and quality-checked using a Qubit 2.0 Fluorometer (Invitrogen) with the Qubit RNA Assay Kit (Molecular Probes), a NanoDrop 1000 Spectrophotometer (Thermo Scientific) and a 2100 Bioanalyzer (Agilent Life Sciences). All the RNAs displayed a RIN above 7.

### Library Preparation and Sequencing

Libraries were prepared from 3 μg of total RNA using the SMARTer Stranded RNA-Seq kit (Clontech). The isolation of mRNAs was performed using the Illumina beads and the TruSeq protocol (Illumina). The final elution of the mRNAs was performed using the Illumina elution buffer (19.5 μl). The isolated mRNAs were quantified using a Qubit fluorometer, as described above. Ten nanograms of mRNA were used for the cDNA synthesis and shearing, following the manufacturer’s instruction. Indexing was performed using the Illumina indexes 1–12. The enrichment step was carried out using 12 cycles of PCR. Subsequently, libraries were checked using a 2100 Bioanalyzer (DNA High sensitivity Kit) to evaluate the mean fragment size. Quantification was performed using the KAPA library quantification kit (KAPA Biosystems) using a ViiA7 Real-Time PCR System (Life Technologies). The pooled libraries (20 pM) were sequenced on an Illumina MiSeq in six consecutive runs (MiSeq reagent kit V3, 150 cycles) generating 76 base pairs (bps) paired-end reads. Raw sequences have been deposited at the NCBI Gene Expression Omnibus (GEO), http://www.ncbi.nlm.nih.gov/geo, accession number: GSE85144.

### Assembly, Mapping, and Data Analysis

Raw sequences reads were uploaded in CLC Genomics Workbench 8.0.3. Sequences were filtered and trimmed as follows: sequence length > 55 bps, sequence quality score < 0.01, no ambiguity in the sequence, trimming using Illumina adaptors, hard trim of 10 bps at the 5′ end and 3 bps at the 3′ end, resulting in a final sequence average length of 61 bps. Duplicated reads were removed from each library using the duplicate read removal plugin. The *de novo* assembly was performed with a wording size ranging from 20 to 54. The reads were mapped back to the assembly with a mismatch, insertion and deletion cost of 3, a coverage > 0.8 and similarity > 0.95. The optimal parameters were obtained with the auto-wording mode (24), bubble sizing in automatic mode. The assembly was then annotated using Blast2GO PRO version 3.0 against the *A. thaliana* non-redundant database. For each library, the mapping was performed with CLC Genomics Workbench 8.0.3 according to the following criteria: a maximum hit per reads of 3, similarity fraction > 0.95, a length fraction > 0.7, a mismatch, insertion and deletion cost of 3. Mapping (using the same above-described parameters) was also performed using the available transcriptome for the variety Finola (van Bakel et al., 2011), which was annotated using Blast2GO PRO version 3.0 against the *A. thaliana* non-redundant database. The expression values were then calculated using the RPKM method (Mortazavi et al., 2008). Genes with less than 10 mapped reads, with no specifically mapped reads in at least one of the libraries, were removed from the dataset. In order to highlight the differentially expressed genes, an ANOVA with four groups (H6, H9, H15, and H20) composed by three biological replicates was performed. A false discovery rate (FDR) correction was then applied to the dataset and genes with FDR corrected *p*-value below 0.05 were selected. Hard cut-offs were finally performed on the fold change (FC absolute value > 2) and the RPKM minimum difference (RPKM difference > 10).

Independent component analysis (ICA) was performed to assess the differences between the biological replicates within the online service MetaGeneAlyse v1.7.1 (Daub et al., 2003). MapMan functional annotations of H6 and H20 were performed with a subset of genes (-2 > Fold Change (FC) > 2) showing a TAIR ID. GOE analysis of H6 and H20 was realized with genes displaying -2 > log2 FC > 2 using ClueGO (v2.1.1) and CluePedia (v1.1.1) (Bindea et al., 2009, 2013) within Cytoscape (v3.2.1) with the following parameters: gene ontology from level 3 to level 9, using All_Experimental_evidence, kappa score set at 0.2, Benjamini–Hochberg correction. The presence of a signal peptide cleavage site in the proteins presumably excreted to the wall was determined with PrediSi^1^.

### RT-qPCR Validation

The RNA-Seq data were validated with RT-qPCR using a subset of 10 genes involved in cell wall biogenesis. Reverse transcription was carried out with the ProtoScript II Reverse Transcriptase (NEB) following the manufacturer’s instructions. Primers were designed with Primer3 and validated for the absence of dimers and secondary structures (hairpin) using OligoAnalyzer 3.1^2^. qPCR runs were performed in 384 well-plates, on a ViiA7 Real-Time PCR System (Applied Biosystems) with the Takyon SYBR Green low ROX (Eurogentec). A melt curve was realized at the end of each experiment to ensure the specificity of the products. Relative gene expressions were determined with the qBase^PLUS^ software v2.5 (Biogazelle). The geNorm analysis (Vandesompele et al., 2002) designated *CsaETIF4E* and *CsaGAPDH* as the most suitable genes for normalization (among *Histone, EF2, Actin, Cyclophilin, Ubiquitin, GAPDH, Tubulin, ETIF4E, ETIF3H*, and *ETIF3E*). Calibrated Normalised Relative Quantities (CNRQ) from RT-qPCR were used for the final comparison with the RPKM values.

### Phytohormones Quantification

The concentrations of phytohormones were determined with the method of Dobrev and Vankova (2012) using technical duplicates on biological triplicates (in H6, H9, H15, and H20). Briefly, 5–10 mg of lyophilised material was extracted with methanol/formic acid/water (15:1:4; v/v) with prior addition of 10 pmol [^2^H]-labeled internal standards described previously (Djilianov et al., 2013). Extracts were evaporated in vacuum concentrator and purified through mixed mode reversed phase – cation exchange SPE column (Oasis-MCX, Waters). The first fraction containing ABA, auxins and JAs and the second fraction containing cytokinins were concentrated to dryness prior to LC-MS. An aliquot (10 μl) of purified sample was analyzed on LC-MS consisting of HPLC (Ultimate 3000) coupled to hybrid triple quadrupole/linear ion trap mass spectrometer (3200 Q TRAP) set in selected reaction monitoring mode. The quantification of hormones was done using isotope dilution method with multilevel calibration curves. Data processing was carried out with Analyst 1.5 software (Applied Biosystems).

### Resin-Embedded Microscopy and Immunohistochemistry

Hemp hypocotyls were embedded in Technovit 7100 resin (Kulzer). Briefly, sections of 5 mm were fixed in glutaraldehyde/paraformaldehyde/caffeine (1%/2%/1% v/v in Milli-Q water) under vacuum for 15 min and 24 h at 4°C, dehydrated in an ethanol series (70–95–100%), impregnated in resin containing PEG 400 (2% v/v) and dimethacrylate ethylene glycol (0.4% w/v) and finally included. Cross sections of 10 μm thickness were cut using a microtome (Leica) and stained with toluidine blue or used for immunohistochemistry (IHC). Image acquisition was performed with a Leica DMR for toluidine blue and with a confocal microscope LSM 510 Meta (Zeiss) for IHC.

LM5 (β-1,4-galactan), LM10 (xylan) and LM15 (xyloglucan) (Plant Probes) antibodies were diluted 10-fold in milk protein (MP)/PBS (5% w/v). Sections were then incubated for 1.5 h, rinsed three times in PBS and incubated for 1.5 h with the anti-rat IgG coupled to FITC (Sigma) diluted 100-fold in MP/PBS. Before observation, three washing steps with PBS were performed. CBM3a (crystalline cellulose, Plant Probes) was diluted to 10 μg/mL in MP/PBS, incubated in mouse anti-His monoclonal antibody (1% in MP/PBS, Sigma) and finally incubated in 50-fold diluted anti-mouse IgG coupled to FITC (Sigma). Each incubation lasted for 1.5 h. Between each step, three washes with PBS were performed. The slides were mounted in Möwiol 4-88 (Sigma) and observed with the following settings: excitation at 488 nm, filter HFT 488/594 and emission recorded with LP 505. The microscope settings were kept rigorously constant between the different observations for a given epitope. Negative controls where either the primary or secondary antibody was omitted resulted in a very weak and negligible signal.

## Results

### Cell Wall Fingerprinting of the Growing Hemp Hypocotyl

**FIGURE 1:**
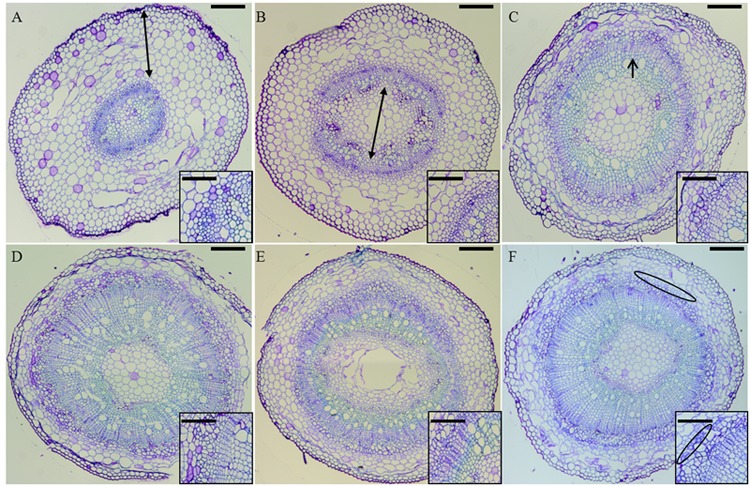
**Cross-sections of H6** (**A**, top left), H9 (**B**, top center), H12 (**C**, top right), H15 (**D**, bottom left), H17 (**E**, bottom center), and H20 (**F**, bottom right) stained with toluidine blue. In **(A)**, the double-pointed arrow spans the cortical parenchyma. In **(B)**, the double-pointed arrow spans the medullary parenchyma. In **(C)**, the arrow points to the developing xylem. In **(F)**, the phloem bast fibers are circled in black. Scale bar: 200 μm in the main pictures, 50 μm in the insets. The figure insets show zoomed regions of the hypocotyls where bast fibers gradually differentiate.

In order to study the development of the secondary tissues in hemp, hypocotyls aged between 6 (H6) and 20 days (H20) were studied. The development of stem tissues during development was observed on hypocotyl cross sections stained with toluidine blue (**Figure 1**). In H6 the cortical parenchyma constitutes the bulk of the section, with collenchyma acting as supporting tissue and medullar parenchyma occupying a limited area (**Figure 1A**). In H9 the cambium has started multiple divisions, with the primary xylem being more developed. Simultaneously, the cells of the medullary parenchyma have grown, leading to an equilibrium between this tissue and cortical parenchyma (**Figure 1B**). At this point, the hypocotyl has stopped its elongation and secondary growth takes place (Supplementary Image S1). In H12, vessels of the secondary xylem are visible (**Figure 1C**). In H20, bundles of phloem fibers are well visible (**Figure 1F**). Between H15 and H20 (**Figures 1D–F**), no major changes occur, secondary xylem being the most prominent tissue and phloem bast fibers with thicker gelatinous walls getting distributed regularly between the cambial zone and the cortical parenchyma.

Confocal microscope observations were carried out on four representative time points, i.e., H6, H9, H15, and H20. Monoclonal antibodies recognizing β-1,4-D-galactan (LM5), xyloglucan (LM15), and xylan (LM10) and His-tagged recombinant protein recognizing crystalline cellulose (CBM3a) were used to study the distribution of key cell wall components during hemp hypocotyl development. We decided to use these specific antibodies to: (1) be able to differentiate primary and secondary growth using “marker” cell wall components (in these specific case hemicelluloses, i.e., xyloglucan and xylan) and (2) to provide a visual inspection of the bast fiber development (by following cellulose accumulation).

**FIGURE 2:**
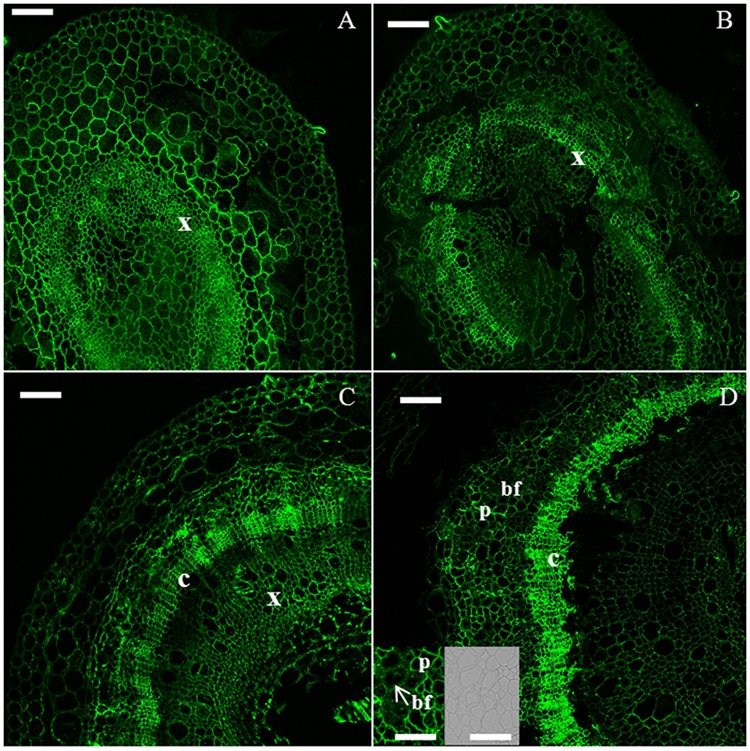
**Immunodetection of the LM5 epitope specific for β-1,4-D-galactan.** H6 (**A**, top left), H9 (**B**, top right), H15 (**C**, bottom left), H20 (**D**, bottom right). bf, bast fiber; c, cambium; p, parenchyma; x, xylem. Scale bar: 100 μm in the main pictures and 50 μm in the insets. The inset shows a zoomed detail of the cortex, where bast fibers are visible. For clarity of presentation, the corresponding DIC image is provided in **(D)**.

Galactan was detected in cambial cells and young xylem cells in H6 and H9 (**Figures 2A,B**), as well as in the parenchyma surrounding developing bast fibers in H15 and H20 (**Figures 2C,D**). The bast fibers did not show labeling (**Figure 2D**, inset).

**FIGURE 3:**
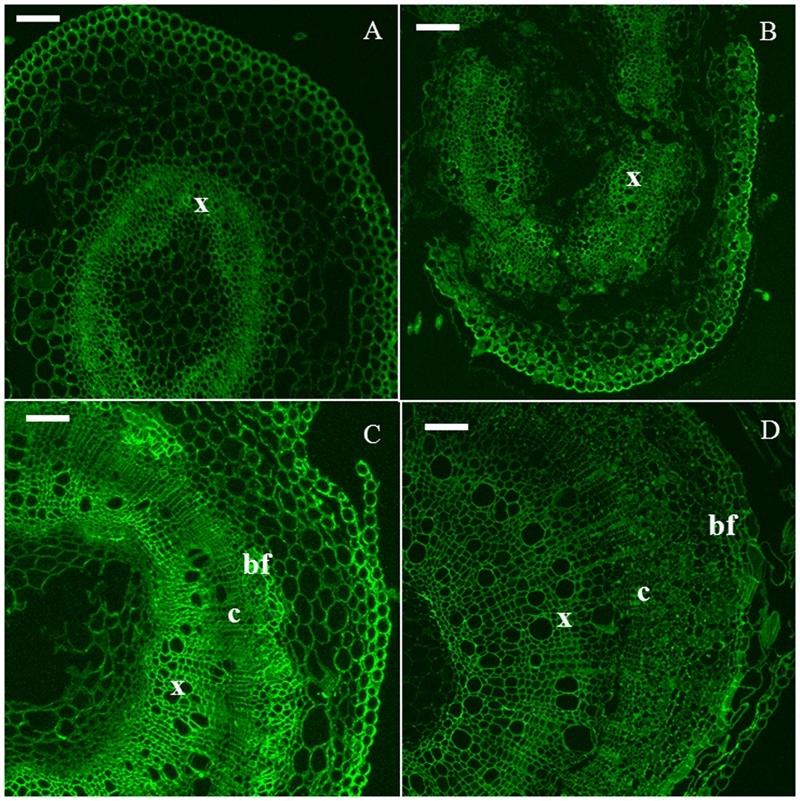
**Immunodetection of the LM15 epitope specific for xyloglucan.** H6 (**A**, top left), H9 (**B**, top right), H15 (**C**, bottom left), H20 (**D**, bottom right). c, cambium; bf, bast fiber; x, xylem. Scale bar: 100 μm.

The xyloglucan epitope was detected in all the time points (**Figure 3**). In H6 and H9 (**Figures 3A,B**), the strongest signal was observed in developing xylem cells. In H15, the young primary bast fibers displayed an intense signal (**Figure 3C**). In older bast fibers, this signal decreased, leading to a comparable intensity in all the tissues of H20 (**Figure 3D**).

**FIGURE 4:**
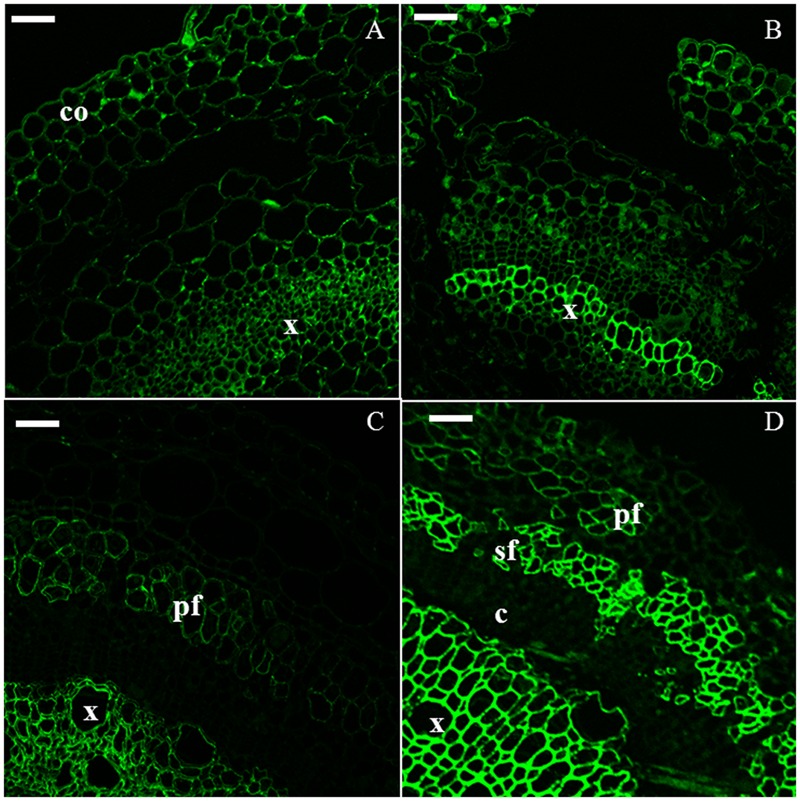
**Immunodetection of the LM10 epitope specific for xylan.** H6 (**A**, top left), H9 (**B**, top right), H15 (**C**, bottom left), H20 (**D**, bottom right). c, cambium; co, collenchyma; pf, primary bast fiber; sf, secondary bast fiber; x, xylem. Scale bar: 50 μm.

The xylan epitope was associated with the xylem of H6 and H9 (**Figures 4A,B**). In H15, xylem cells were heavily stained and a weaker signal was detected in the cell wall of primary bast fibers (**Figure 4C**). In H20, secondary bast fibers displayed a signal which was more intense than the one detected in the primary bast fibers (**Figure 4D**).

**FIGURE 5:**
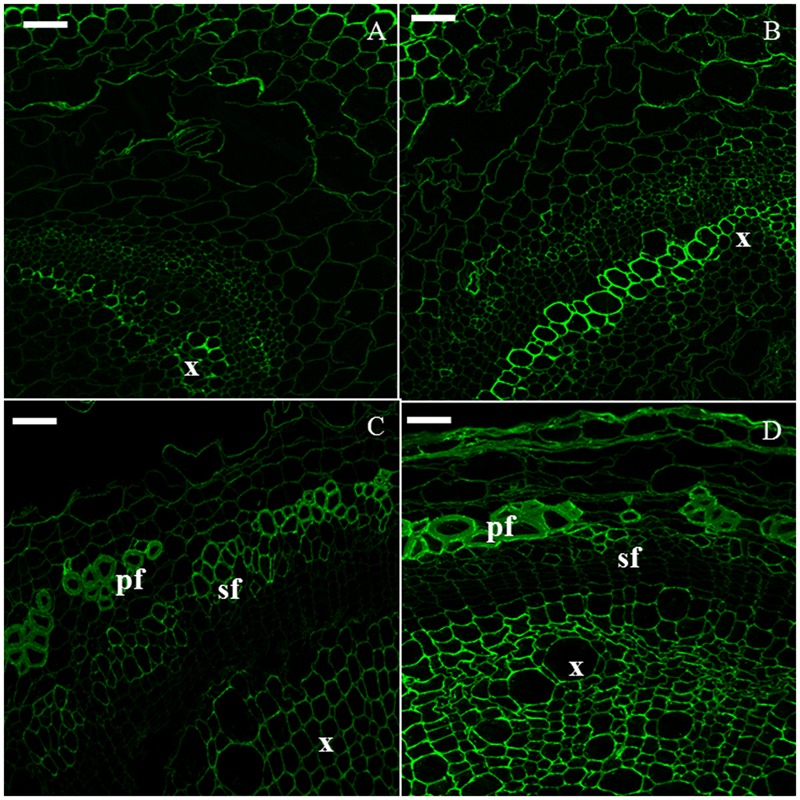
**Immunodetection of the CBM3a epitope specific for crystalline cellulose.** H6 (**A**, top left), H9 (**B**, top right), H15 (**C,** bottom left), H20 (**D**, bottom right). pf, primary bast fiber; sf, secondary bast fiber; x, xylem. Scale bar: 50 μm.

Crystalline cellulose was visualized with CBM3a (**Figure 5**). It was observed in all the tissues at each time point, particularly in xylem cells in H6 and H9 (**Figures 5A,B**) and in bast fibers in H15 (**Figure 5C**) and H20 (**Figure 5D**). Indeed, in the older hypocotyl, the gelatinous walls of both primary and secondary bast fibers were strongly labeled by the antibody.

### Transcriptome Overview of the Growing Hemp Hypocotyl

In order to get a detailed picture of the gene expression changes during the development of the hemp hypocotyl, RNA-Seq was performed on four representative time points (H6, H9, H15, and H20). As can be seen in **Figure 1**, these time-points correspond to key developmental stages of the hemp hypocotyl (elongation and secondary growth, development of bast fibers). After filtering and trimming, duplicated reads were removed (4–7% of the filtered reads). The sequences were assembled into 28433 contigs with size ranging from 282 to 15600 bps, an average length of 1136 bps and a N50 of 1660 bps (Supplementary Table S1). 84–88% of the reads were successfully mapped to the Santhica assembly with more than 99% of reads specifically mapped (Supplementary Table S1). After an ANOVA statistical analysis, 3622 differentially expressed contigs were selected. When mapping against the Finola assembly (van Bakel et al., 2011), 3096 contigs were differentially expressed (Supplementary Table S2).

The RNA-Seq results were verified by quantifying the gene expression levels of 10 representative genes by RT-qPCR (Supplementary Table S3). Genes involved at different levels of cell wall biogenesis, displaying significant RPKM fold change between the time-points and covering a large range of expression (from 3 to 1600+ RPKM), were chosen. A good correlation was obtained between the RNA-Seq and RT-qPCR data (*R*^2^ = 0.91).

**FIGURE 6:**
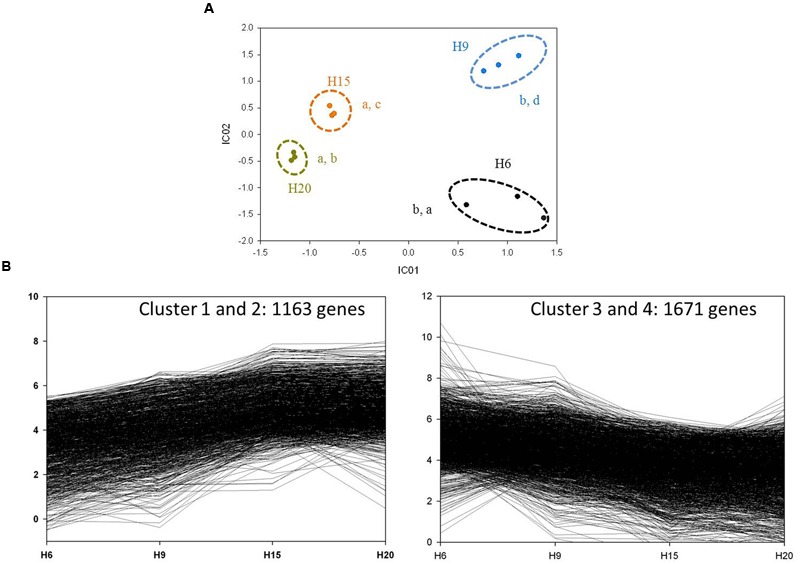
**General analysis of H6, H9, H15, and H20 transcriptomes. (A)** ICA of H6 (black dots), H9 (blue dots), H15 (orange dots), and H20 (dark yellow dots). Variance explained by the two independent components: 89.36%. The three biological replicates of each time point are surrounded by a dotted line. Letters correspond to homogeneous groups determined by the Tukey *post hoc* test on IC1 and IC2 coordinates, respectively. **(B)** Clusters with up-regulated and down-regulated genes through the time-points.

Independent Component Analysis (Daub et al., 2003) performed on RPKM values resulted in a good separation between each time-point when using two independent components (**Figure 6A**). The expression profiles of the 3622 differentially regulated genes were assessed by hierarchical clustering using a Euclidean distance matrix in complete linkage. Thirteen clusters were obtained (Supplementary Image S2), with the four most important (C1–C4) accounting for 81% of the genes (**Figure 6B**). Genes up-regulated at 15 and 20 days were mainly distributed in C1 and C2. The transcripts more abundant at 6 and 9 days were distributed in C3 and C4 (**Figure 6B**).

### Transcription Factor Inventory of the Growing Hemp Hypocotyl

We looked for differentially expressed genes coding for putative transcription factors (TFs) in hemp by searching for their *Arabidopsis* orthologs (Jin et al., 2014). A total of 131 contigs were obtained (Supplementary Table S4). Most of these contigs (34%) were more expressed in H6, with proportions decreasing along time (26, 23, and 17% for H9, H15, and H20, respectively). The basic helix-loop-helix (bHLH) family was the most represented across the time-points studied, with 23 members, followed by the homeobox domain (HB) and C2H2 (12 members each).

We could discriminate between TFs expressed at earlier and later time-points and hence get a TF signature of the developing hemp hypocotyl. Following emergence from the soil, several TFs related to circadian rhythm, response to light, photosynthesis, pigment biosynthesis, cell expansion, and vascular development were more abundant in H6 and H9 (Supplementary Image S3). More specifically, members of the HB, bHLH, C2H2, and MYB families were abundantly expressed in H6 and H9 (Supplementary Table S4).

The transcriptome of the older hemp hypocotyl was characterized, as expected, by a more abundant expression of TFs related to SCW biosynthesis and vascular development (Supplementary Table S4). Three master regulators of SCW deposition, namely *SND2, VND1*, and *NST1* displayed the highest expression in H15, together with a second-layer master switch, *MYB46*, involved in SCW biogenesis and xylan biosynthesis (Zhong et al., 2008). *XND1* was more abundant in H20. As far as the TFs regulating cell wall composition are concerned, a higher expression of TFs involved in hemicellulose and lignin biosynthesis were found in H20, i.e., *KNAT7* and *MYB85*. An ortholog of *Arabidopsis WLIM1*, involved in fiber extension and lignification, was more abundant in H20 (Supplementary Table S4).

### Hemp Hypocotyl Transcriptome Dynamics as Seen from a Cell Wall Angle

Based on the general analysis of their transcriptomes, one can separate H6 and H9 from H15 and H20. For further characterisation of the hypocotyl transcriptome dynamics, a focused comparison between H6 and H20 will hereby be made.

**FIGURE 7:**
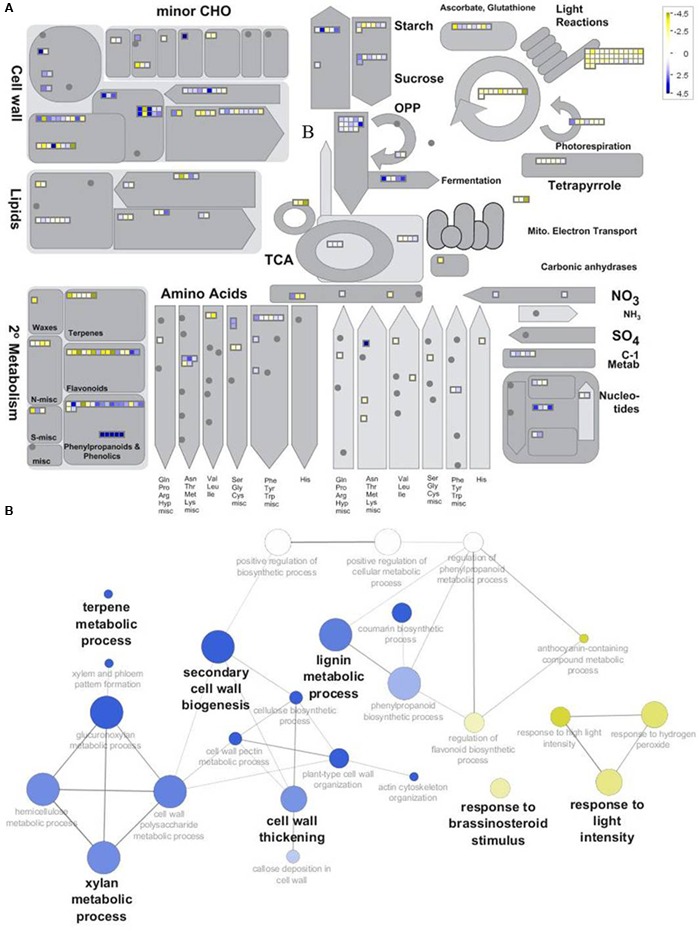
**Overview of the differentially expressed genes between H6 and H20. (A)** Top: Metabolism overview provided by MapMan functional annotation. Genes up-regulated in H6 and H20 are represented by *yellow* and *blue* squares, respectively. **(B)** Bottom: GOE performed with the subset of genes showing -2 > log_2_FC (H20/H6) > 2 in Cytoscape using ClueGO and CluePedia. Biological processes with genes overrepresented in H6 and H20 are shown in *yellow* and *blue*, respectively.

As a result, from the 3622 transcripts used to build the ICA and clustering (**Figure 6**; Supplementary Image S2), those associated with an *Arabidopsis* locus ID and showing a difference corresponding to -1 > log_2_ FC (H20/H6) > 1 were used to compare H6 and H20. MapMan functional annotations highlighted the differences between these two time points (**Figure 7A**; Supplementary Table S5). Seven categories showed significant differences (*p*-value < 0.05): photosynthesis, cell, cell wall, gluconeogenesis/glyoxylate cycle, glycolysis, nucleotide metabolism, fermentation (Supplementary Table S5). In addition, a subset of the most differentially expressed contigs (-2 > log_2_ FC (H20/H6) > 2) have been represented using ClueGO and CluePEDIA within Cytoscape (**Figure 7B**). Genes important for cell wall biogenesis are listed in Supplementary Table S6.

As the hypocotyl develops and builds secondary structures, its cellular organization undergoes dramatic changes, as shown by the expression changes of those genes involved in cell wall biosynthesis and assembly (Supplementary Table S6; Supplementary Image S4).

In H6, elongating cells are surrounded by the PCW, while later vascular and sclerenchyma tissue development is characterized by secondary growth and associated SCW deposition. To highlight the dynamism existing at the cell wall level, a description of the evolution of five families of enzymes playing a predominant role in matrix polysaccharide biosynthesis and remodeling is hereby made following the CAZy nomenclature (Lombard et al., 2014). The families described are the CSL (GT2 family), the β-galactosidase (GH35 family), PME (CE8 family), expansin (CBM 63 family), and XTH (GH16 family). A separate section is devoted to the analysis of those genes involved in SCW biosynthesis.

At the transcriptional level, PCW deposition and organization depend on a large number of factors. PCW is mainly composed of cellulose deposited in parallel arrays, hemicellulose (predominantly xyloglucan) and pectins (Park and Cosgrove, 2015). The modification of the CW during plant development relies on the active remodeling of the matrix polysaccharides. The RNA-Seq analysis has shown that in H6 genes belonging to the CSL C and E families were more abundantly expressed with respect to H20 (Supplementary Table S6). More specifically, *CSLC4* and *CSLE1* were more abundant in H6. In H20 different members of the CSL family were highly expressed, namely *CSLD3* and *CSLD5*.

Members of the GH35 and CE8 families were also differentially expressed between H6 and H20 (Supplementary Table S6). The RNA-Seq analysis showed a differential expression of β-galactosidase (GH35) members in H6 and H20. In H6, the orthologs of *Arabidopsis* β*GAL2* and β*GAL10* were more abundant. In H20, the ortholog of β*GAL3* was instead more abundant. Pectin methyl esterases (PMEs) and polygalacturonases (GH28 family) were essentially more expressed in H20 (e.g., orthologs of At5g47500 and At5g63180), with only a few being more expressed in H6 (e.g., orthologs of At5g20860 and At4g23820). DUF642, an activator of PME activity (Zúñiga-Sánchez et al., 2014), was strongly up-regulated in H6. Two orthologs of *Arabidopsis* expansins, *EXPA5* and *EXPA8*, were more abundant in the young hypocotyl, as well as three XTHs. *EXPA4* was instead more expressed in H20.

Xyloglucan endotransglucosylase/hydrolase are divided into two categories: XEHs and XETs. XEHs irreversibly shorten the length of the chain, while XETs ligate and cleave xyloglucan chain coming from the Golgi (Eklöf and Brumer, 2010). The only XEH (*XTH31*) retrieved in our study was more abundant in H6; some XTHs were more abundant in H6 (*XTH5, XTH8*), while others (*XTH15, XTH22*) were preferentially expressed in H20.

### Secondary Cell Wall-Related Genes

Secondary cell wall biogenesis requires the fine tuning of numerous metabolite biosynthesis which are deposited in specific tissues (e.g., xylem vessels and sclerenchyma) undergoing lignification. As expected, several genes linked to SCW formation were more abundant in H20 as compared to H6 (Supplementary Table S6; Supplementary Image S4).

Among the SCW-related genes up-regulated at later stages of development were the orthologs of *A. thaliana CesA4, CesA7* and *CesA8* (Supplementary Table S6), responsible for the deposition of cellulose. Several genes related to the cellulose synthase complex trafficking were more abundant in H20 (Supplementary Table S6). Such genes included orthologs of *MAP70-5* and *MAP65-8, tubulin* β*-2, tubulin* β*-8* and several genes related to the microtubule motor activity (kinesin motor domain, ATP binding microtubule motor family protein).

Several transcripts related to xylan biosynthesis and xylan export were more abundant in H20, notably two isoforms of UDP-xylose synthase, *UXS2* and *UXS5*, which provide the building blocks for polymerisation into the xylan backbone. Genes involved in glucuronoxylan synthesis (the main xylan in dicots; Zhong and Ye, 2015) were more abundantly expressed at later stages of development. Other transcripts involved in xylan biosynthesis and more expressed in H20 include *CSLD5, TBL34, DUF547, ESK1*, and *DUF579*. A more pronounced *trans*-Golgi network activity at later growth stages was highlighted by the higher expression of *DUF707, DUF821* and a clathrin light chain protein at H20 (Supplementary Table S6).

H20 expression pattern exhibited a substantial up-regulation of the genes associated with lignin biosynthesis. In the general phenylpropanoid pathway, orthologs of *Arabidopsis PAL1, PAL2, C4H, 4CL1*, and *4CL2* were 1.88 to 2.98 fold more expressed (log_2_FC) in H20 than in H6. In the monolignol pathway, orthologs of *Arabidopsis HCT, CCR1, CCoAOMT*, and *CAD4* were up-regulated from 1.58 to 2.79 (log_2_FC) (Supplementary Table S6). In relation to the polymerisation of the monolignol subunits, five orthologs of *Arabidopsis* laccase isoforms (LAC) were dramatically induced in H20: *LAC4* (log_2_ FC 5.69), *LAC5* (log_2_ FC 4.85), *LAC12* (log_2_ FC 4.32), *LAC13* (log_2_ FC 5.02) and *LAC17* (log_2_ FC 5.75). Complementary to the laccase-driven lignin polymerisation, class III peroxidases were also differentially expressed between H6 and H20. The ortholog of *AtPRX52*, which is involved in the synthesis of S-lignin in fibers (Fernández-Pérez et al., 2014), was more abundant in H20. The hemp ortholog of *AtPRX64*, another peroxidase involved in fiber lignification (Tokunaga et al., 2009), was also highly expressed in H20 (Supplementary Table S6).

### Endogenous Phytohormones Content at Different Stages of Development

**FIGURE 8:**
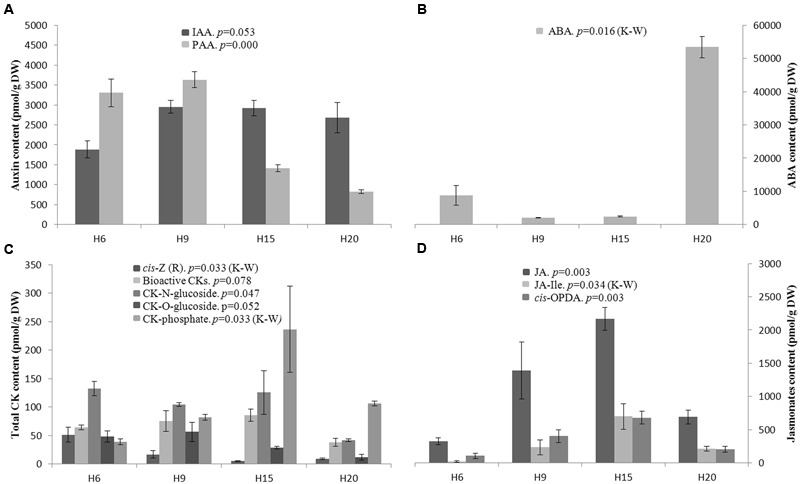
**Endogenous phytohormone levels in hemp hypocotyls at H6, H9, H15, and H20. (A)** (top left): bioactive auxins; IAA indole-3-acetic acid, PAA phenylacetic acid. **(B)** (top right): ABA, abscisic acid. **(C)** (bottom left): Total cytokinin. **(D)** (bottom right): bioactive JAs; JA, jasmonic acid; JA-Ile, jasmonoyl-isoleucine; *cis*-OPDA, *cis*-(+)-12-oxo-phytodienoic acid. Mean values ± SE are given. ANOVA or Kruskal–Wallis (K–W) results are indicated depending on the normality of data distribution (Shapiro–Wilk *p* > 0.05 or *p* < 0.05, respectively). *n* = 3 biological replicates.

Several phytohormones belonging to the auxin, abscisic acid (ABA), cytokinin (CK) and jasmonate (JA) families were quantified. The indole-3-acetic acid (IAA) content was not statistically different among the time-points, while the phenylacetic acid (PAA) content was significantly higher in H6 and H9 as compared to H15 and H20 (**Figure 8A**).

Abscisic acid content was remarkably higher in H20 as compared to H6, H9, and H15 (**Figure 8B**). When considering the total content of bioactive CKs, no significant differences were found (**Figure 8C**). The *cis*-zeatin and *cis*-zeatin riboside content was higher in H6. According to Havlová et al. (2008), *cis*-zeatin and its riboside lack physiological activity in most CK bioassays and was therefore not considered as an active form of CK. The CK N-glucoconjugates (deactivation forms) and *O*-glucoconjugates (storage forms) levels were almost equal in H6, H9, and H15 with a decrease in H20 (**Figure 8C**). By contrast, the CK-phosphates (CK biosynthesis intermediates) content was higher in H15 and H20 (**Figure 8C**). The bioactive forms of JAs include jasmonic acid (JA), jasmonoyl-isoleucine (JA-Ile) and *cis*-OPDA. Both JA and *cis*-OPDA contents were significantly higher in H15 than in H6 (**Figure 8D**).

**FIGURE 9:**
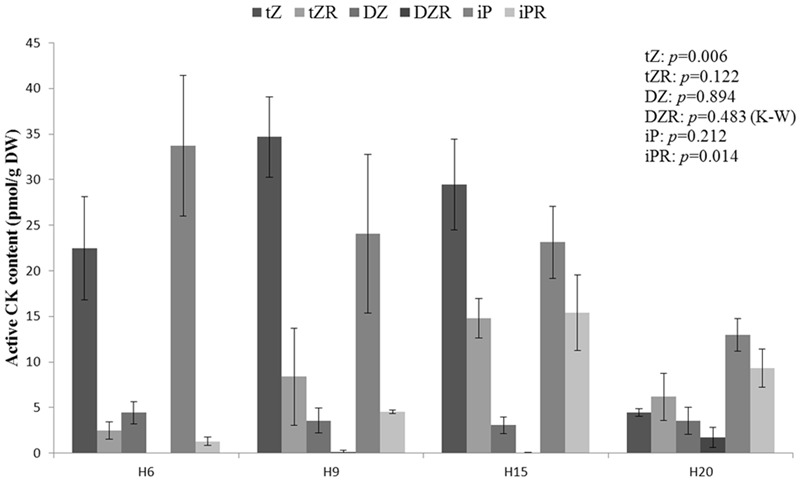
**Endogenous bioactive cytokinin levels in hemp hypocotyls at H6, H9, H15, and H20.** tZ, *trans*-zeatin; tZR, *trans*-zeatin riboside; DZ, dihydrozeatin; DZR, dihydrozeatin riboside; iP N6-(Δ2-isopentenyl)adenine, iPR N6-(Δ2-isopentenyl)adenosine. ANOVA or Kruskal–Wallis (K–W) results are indicated depending on the normality of data distribution (Shapiro–Wilk *p* > 0.05 or *p* < 0.05, respectively). *n* = 3 biological replicates.

The bioactive portion of CK pool is shown in **Figure 9**. The variation described in **Figure 8C** was due to the significant difference observed in the content of *trans*-zeatin (tZ) and its riboside. The *trans*-zeatin/riboside content was higher in H9 and H15 with a minimum in H20 (ANOVA and Tukey *post hoc* test, *p* = 0.014). No significant differences were found in the contents of dihydrozeatin (DZ) and its riboside, which were lower as compared to the other forms of bioactive CKs. Even if this trend was not statistically significant, the *N*^6^-(Δ^2^-isopentenyl)adenine (iP) pool decreased constantly between H6 and H20. The corresponding riboside iPR transiently peaked in H15. The ratios tZ/tZR, DZ/DZR, and iP/iPR were higher in the young stages of development and decreased in older hypocotyls. However, only the tZ/tZR and iP/iPR ratios were found to be significantly different between the time-points (ANOVA *p*-values of 0.035 and 0.008, respectively).

## Discussion

In this study, we have performed immunohistochemistry, analyzed the transcriptome and quantified key phytohormones in the hemp hypocotyl to analyze the events associated with secondary growth and bast fiber development. By using hypocotyls aged between 6 and 20 days, we could obtain a snapshot of the cell wall-related events accompanying the shift from primary to secondary growth and bast fiber formation in a cultivar of textile hemp (Santhica 27, Supplementary Image S4). The discussion will point to the main findings of this study regarding the primary and secondary growth of the hemp hypocotyl, the differential regulation of the phenylpropanoid-related genes and the interactions between the phytohormones and the cell wall-related genes.

### Regulation of the Elongation in the Hemp Hypocotyl

The hypocotyl elongation is under the control of TFs and genes involved in both cell wall deposition and modification. The bHLHs TFs *KDR* and *AIF3* have antagonistic actions in cell elongation (Ikeda et al., 2013) and were both more expressed in H6 (Supplementary Table S3). Another elongation repressor of the bHLH system, *IBH1*, was more expressed in H15 and H20 as compared to H6 and H9 (Ikeda et al., 2013). *LHW* is related to vascular development (De Rybel et al., 2016) and was more abundant in H9. A TF with a dual function on cell wall dynamics showed an interesting expression pattern, namely *WLIM1*. In cotton fibers, WLIM1 bundles the actin filaments, which favors fiber extension by activating intracellular transport. It also binds to the PAL-box in the promoters of lignin/lignin-like biosynthetic genes, thereby contributing to the lignification of the fiber SCW (Han et al., 2013). The role of WLIM1 in fiber extension can be proposed in young hemp hypocotyls, since a significant increase in expression was observed between H6 and H9 (Supplementary Table S4).

Xyloglucan, a hemicellulose of the PCW, plays a crucial role in the regulation of cell elongation (Park and Cosgrove, 2015). It was detected in the cell wall at all the time points studied (**Figure 3**) and *CSLC4*, which is involved in its biosynthesis, was more expressed in H6. CSLC4 acts in the Golgi to form the xylosylated glucan backbone during xyloglucan biosynthesis (Park and Cosgrove, 2015). Several transcripts related to xyloglucan remodeling were detected in H6, including expansins, XTH, and β-galactosidases. XTH and expansins transcripts were similarly found in elongating flax hypocotyls (Roach and Deyholos, 2008). Expansins loosening the cell wall are thought to be active in ‘biomechanical hotspots’ where xyloglucan and cellulose are closely intertwined (Park and Cosgrove, 2015). *XTH31* was more expressed in the actively growing hypocotyl (H6), suggesting its potential role in cell expansion (Franková and Fry, 2013). In elongating tissues, β*GAL10* may have an activity on xyloglucan (Sampedro et al., 2012). Cell wall loosening is also controlled by peroxidases (Francoz et al., 2015), which break covalent bonds in cell wall polymers. Notably, in our dataset, the ortholog of *AtPRX57* was strongly upregulated at H6 (Supplementary Table S6). The deduced protein sequence showed the presence of a signal peptide, a feature supporting the potential role of this peroxidase in cell wall loosening during the growth of the young hemp hypocotyl.

### Differential Expression of Genes in the Phenylpropanoid Pathway among Time Points

Anthocyanin biosynthesis and monolignol production share the phenylpropanoid pathway from phenylalanine to *p*-coumaroyl CoA. The latter is either directed to the flavonoid pathway through CHS, or through the monolignol pathway via CCR- or HCT- mediated reactions. Several genes implicated in anthocyanin biosynthesis were more expressed in H6 (*MYB114, CHS*, and *TT18*) while those involved in monolignol biosynthesis were more expressed in H15 or H20 (*HCT, CCoAOMT*; Supplementary Table S6). Anthocyanins modify the quantity and quality of light trapped by the chloroplasts, acting as a photoprotectant (Steyn et al., 2002). This photoprotection is a required adaptation when the hypocotyl emerges from the soil (Wang et al., 2012). Monolignols are incorporated into the lignin polymer at older developmental stages to provide strength and resistance to the conducting and supporting tissues. A recent work has shown that anthocyanin and monolignol biosynthesis may be interdependent. Indeed, the downregulation of *CCoAOMT*, a core enzyme of the monolignol biosynthetic reactions, activates the anthocyanin pathway through a MYB TF in petunia (Shaipulah et al., 2016). In addition, silencing of *HCT* in *Arabidopsis* was shown to decrease the S units and increase the H units and flavonoid production through CHS activity (Hao and Mohnen, 2014).

### Marker Genes of the Hemp Hypocotyl Secondary Growth

The cambial activity marking the onset of secondary growth is controlled by phytohormones and, notably, active JAs (Sehr et al., 2010). The JAs content peaked at H15 (**Figure 8**), where cambial division is clearly visible (**Figure 1D**). JAs stimulate secondary growth by enhancing cambial activity (Sehr et al., 2010) and favor both phloem fiber formation (Sehr et al., 2010) and lignification (Pauwels et al., 2008). At the transcriptional level, the expression of the master regulator of fiber differentiation *NST1* (Zhong and Ye, 2015) increased sixfold in H15 as compared to H6 (Supplementary Table S4). XND1 is another NAC TF which acts antagonistically to NST1 to ensure xylem extensibility (Zhao et al., 2008) and its expression increased 23-fold in H20 as compared to H6 (Supplementary Table S4). PXC1 regulates the xylem fiber formation in stem secondary vasculature (Nieminen et al., 2015) and its expression increased threefold in H15 as compared to H6. *WAT1*, encoding a vacuolar auxin transporter essential for fiber differentiation and secondary wall thickening (Ranocha et al., 2013), showed increased expression (2.6-fold) in H20 as compared to H6. *WOX4*, which codes for a homeobox module involved in hypocotyl vascular patterning and cambium proliferation (Ragni and Hardtke, 2014), was more expressed in H20 (Supplementary Table S4). Genes involved in wall remodeling were also differentially regulated during the development of the hypocotyl. For example, the late expression of the ortholog of *AtEXPA4* (Supplementary Table S6) might be involved in secondary vascular tissue development by, e.g., regulating intrusive growth (Gray-Mitsumune et al., 2004). Since hemp bast fibers also grow intrusively (Snegireva et al., 2015), a role for this protein in this process might be hypothesized. This gene was found to be more expressed in the phloem-cambium zone of the *Arabidopsis* hypocotyl undergoing secondary growth (Zhao et al., 2005), where active division and cellular growth occur. The hemp ortholog of *TCH4* (*XTH22*) was strongly upregulated in H20 and is known to be inducible by *cis*-OPDA (Taki et al., 2005). The presence of *TCH4* in tissues undergoing secondary wall deposition may be explained by the function of xyloglucan in the biogenesis of the cell wall. Indeed, xyloglucan, after transit through the SCW, may reinforce the links between PCW and SCW in the S1 layer junction (Bourquin et al., 2002). This may explain the strong LM15 signal observed within the cell wall of H15 bast fibers (**Figure 3C**) and the late up-regulation of *TCH4*.

### Transcripts Involved in the SCW Biogenesis

Our data showed that the genes involved in the biosynthesis of xylan, cellulose, monolignols and in lignin polymerisation were co-expressed in H15 and H20 (Supplementary Table S6). Such a co-expression may result from the regulation of SCW deposition by TFs, i.e., NST1 for the fiber fate (Hao and Mohnen, 2014) and VND1 for vessels (Zhong and Ye, 2014).

In our dataset, the cellulose synthases which were more expressed during secondary growth were the *Arabidopsis* orthologs of *CesA4, CesA7*, and *CesA8*, as already previously observed in adult hemp (De Pauw et al., 2007; van den Broeck et al., 2008). *CesA*s require sucrose synthase (SUS) activity for the provision of the UDP-glucose (Doblin et al., 2002). The *Arabidopsis* orthologs of *SUS4* and *SUS6* were found to be differentially expressed between young and old hypocotyls (Supplementary Table S6). In *Arabidopsis*, SUS6 is present in the vascular tissues of the cotyledons, leaves, petals, anthers, and roots (Bieniawska et al., 2007). The function of SUS4 and SUS6 possibly overlaps, but they may have different patterns of tissular or cellular localisation. These enzymes may be soluble to provide a pool of hexose phosphate for the cellular metabolism or anchored to the plasma membrane to deliver UDP-glucose to synthetize wall polysaccharides including xyloglucan and cellulose (Bieniawska et al., 2007). Two genes of the COBRA family, *COB* and *COBL4*, are also involved in cellulose biosynthesis (Brown et al., 2005). Further characterisation of the genes belonging to this family is needed to understand their contribution and the way they act in the cell wall. As their expression was higher in H20, a role in SCW and gelatinous layer (G-layer) deposition may be postulated. Supporting this hypothesis, Hobson et al. (2010) have described a COBRA-like gene being strongly up-regulated in the bast fibers of the middle part of adult hemp stems as compared to the top and De Pauw et al. (2007) found that *COBL4* was more expressed in the outer tissue of the middle and bottom parts of the stem as compared to the top, where the G-layer is appearing in phloem fibers. In flax it was demonstrated that, after its deposition, the bast fiber galactan layer progressively matures via the assembly of cellulose microfibrils to a crystalline structure (Gorshkova and Morvan, 2006). In flax, this maturation between the galactan-layer and the G-layer requires βGAL1 (Roach et al., 2011). We have found out that a β*GAL* (contig 1317), ortholog of the *Arabidopsis* β*GAL3*, was specifically expressed in hemp hypocotyls undergoing SCW deposition. This gene clusters with flax *Lu*β*GAL1*, which is involved in SCW deposition in flax hypocotyls and stems (Roach and Deyholos, 2007; Roach et al., 2011). It should, however, be noted that in hemp we did not observe an LM5 signal in the bast fibers (**Figure 2**), as previously shown in adult plants by Blake et al. (2008). This is an important difference with respect to flax fiber composition. Another gene which may play an important role in hemp cell wall maturation is a class IV chitinase (contig 13996). It was highly expressed in H9, H15, and H20. In flax, three isoforms of chitinase-like genes, most similar to chitinases of class IV, were highly expressed in the phloem fibers (Mokshina et al., 2014), where they may be involved in the development of the G-layer. A signal peptide was found in the deduced hemp chitinase protein sequence, a finding corroborating its action in the cell wall. Three isoforms of FLAs (*FLA11, FLA12*, and *FLA16*, Supplementary Table S6) were more expressed in H15 and H20. The biological functions of FLAs are still debated (Roach and Deyholos, 2007), however, several lines of evidence point to a role in SCW deposition (Ito et al., 2005; MacMillan et al., 2015). Our data suggest they may indeed be involved in the deposition of the SCW of the fibers (Roach and Deyholos, 2007), since they were more expressed at older developmental stages. AtFLA11 and AtFLA12 are highly active in xylem vessels and fibers and impact cellulose, arabinose, and galactose content in the cell wall (MacMillan et al., 2015). The group containing AtFLA16 has not yet been characterized. In flax hypocotyls, two FLAs were more expressed at 9 and 15 days as compared to 7 days (Roach and Deyholos, 2008).

Xylan is the chief hemicellulose in hemp hypocotyl secondary tissues (**Figure 4**). Indeed, in accordance with Blake et al. (2008), the LM10 epitope was detected in the cell wall of xylem cells and bast fibers. This finding was supported at the transcriptome level by Cytoscape and MapMan analyses (**Figure 7**). In xylem cells, xylan is largely present in the lignifying SCW while in the bast fibers it was localized in the outermost layer enveloping the G-layer. Several genes involved in the biosynthesis of glucuronoxylan were found in our analysis (Supplementary Table S6). Glucuronoxylan contains a xylan reducing-end glycosyl sequence (XRES) which is likely produced by FRA8 and IRX8 (Hao and Mohnen, 2014). Some genes involved in the elongation of the glucuronoxylan backbone (*IRX10, IRX10L, IRX14*, and *IRX15L*), backbone acetylation (*ESK1, RWA3*) and substituent methylation (*GXM3*) have been found in late stages of the hypocotyl development. Determining the molecular structure and the precise deposition site of xylan in bast fibers, e.g., by immunogold labeling coupled to TEM, may shed light on its function (Mikshina et al., 2013). Indeed, Mortimer et al. (2015) have described in *Arabidopsis* a subset of genes involved in the biosynthesis of a specific type of xylan with pentosylated side chains (*PUX5*) which is present in young stems and roots. Interestingly, *IRX10L* and *IRX14* are part of this subset of genes. The *PUX5*-xylan is able to interact with different wall polymers, but with a reduced cross-linking as compared to the xylan of the SCW, to ensure PCW extensibility. It should be noted that IRX10 and IRX15L seem to be implicated in the backbone elongation of SCW xylan (Jensen et al., 2011; Mortimer et al., 2015). Such a differentiation of genes involved in xylan backbone elongation may point to different functions of the xylan deposited in the xylem cells and bast fibers. Further studies are needed to verify this hypothesis.

Most of the genes involved in the transcriptional regulation of lignification, i.e., the methyl donors, genes involved in monolignol biosynthesis and lignin polymerisation were up-regulated either in H15 or H20. *WLIM1, MYB85*, and *NST1* regulate the transcription of the lignin biosynthesizing genes (Zhong and Ye, 2009). C1 metabolism was clearly more intense as the hypocotyls develop, as shown by the up-regulation of one isoform of methylenetetrahydrofolate reductase and methionine synthase and three isoforms of S-adenosylmethionine synthase (orthologs of *Arabidopsis MTHFR2, ATMS1, SAMS2, SAMS3*, and *SAMS4*). *Arabidopsis* seedlings deficient in SAMS3 activity display significantly lower content of lignin in the aerial part (Shen et al., 2002), thus highlighting the possible regulation of lignification by the pool of available methyl donors. In adult hemp stem, the core tissue mainly composed of xylem cells also shows a higher rate of C1 recycling (van den Broeck et al., 2008). The genes putatively involved in phenylpropanoid and monolignol biosynthesis were all more expressed in H15 or H20, with the exception of *CAD9*. In *Arabidopsis*, CAD9 is involved in the biosynthesis of coniferyl alcohol in the young developing stem (Eudes et al., 2006). It is therefore plausible to observe a higher expression of CAD9 in H6. The other CAD isoform, *CAD4*, reached its highest expression in H20. None of these two CAD isoforms have been described in the studies of De Pauw et al. (2007) and van den Broeck et al. (2008). This may be explained by the higher sensitivity of the RNA-Seq for low abundance transcripts (highest RPKM of *CAD9* = 29). Indeed, other genes involved in these pathways display higher RPKM values (>500) and were detected by these studies based on microarrays.

Peroxidases and laccases are enzymes known to polymerise monolignols in *Arabidopsis* (Hao and Mohnen, 2014). The changes associated with the expression of these genes were among the highest observed in our study (Supplementary Table S6). The orthologs of *AtPRX52* and *AtPRX64*, which are involved in *Arabidopsis* fiber lignification (Tokunaga et al., 2009; Fernández-Pérez et al., 2014), were more expressed in H20. Interestingly, a signal peptide is present in the deduced protein sequences of these two peroxidases, suggesting their secretion to the cell wall. Such a signal peptide was also detected in two (AtLAC5 and AtLAC13 hemp orthologs) out of the five laccase isoforms (see Results section), which may be involved in lignin polymerisation in *Arabidopsis*. Indeed, AtLAC4, AtLAC12, and AtLAC17 have a putative role in stem lignification in *Arabidopsis* (Berthet et al., 2012). AtLAC5 and AtLAC13 may be secreted because of the presence of a signal peptide (Turlapati et al., 2011), but their involvement in lignification has not yet been proven; they cluster together, while AtLAC4, AtLAC12, and AtLAC17 are in three separate clusters according to Turlapati et al. (2011).

### Hormonal Control of Cell Wall Biogenesis in the Hypocotyl of *C. sativa*

Several phytohormones are essential for cell wall biogenesis, from the elongation phase to the strengthening of various tissues by the SCW. Many studies have pointed to their roles in the expression of cell wall related genes, either by direct action or by influencing the genes involved in their transcription or signaling (reviewed by Sánchez-Rodríguez et al., 2010; Didi et al., 2015; Nieminen et al., 2015). We have thus combined our transcriptomics data with the quantification of phytohormones and looked for genes involved in cell wall biogenesis known to be regulated by those molecules.

Indole-3-acetic acid and PAA are two distinct bioactive auxins, but they share the same signaling pathway (e.g., TIR1-IAA3 and TIR1-IAA6) and regulate the same auxin-responsive genes (Sugawara et al., 2015). For instance, auxins are known to induce cell elongation via the expansin and XTH activities (Sánchez-Rodríguez et al., 2010). In our dataset, *EXPA5, EXPA8, XTH5*, and *XTH8* were more expressed in H6 and H9, where the endogenous bioactive auxin content (IAA3 and PAA) is higher. Another property of auxin is to prevent lignification by promoting the degradation of IAA3 (Nanao et al., 2014; Oh et al., 2014). In *Arabidopsis* seedlings, the gain-of-function *iaa3/shy2-2* mutant shows higher expression of several genes belonging to the phenylpropanoid/lignin pathway, such as *PAL1, PAL2, C4H, 4CL1, CCoAOMT*, and *PRX52* (Oh et al., 2014). Indeed, the lowest bioactive auxin levels, recovered in H15 and H20, correspond to the highest abundance of these genes. However, auxin may also induce the expression of some genes involved in lignin biosynthesis such as *COMT* and *CCoAOMT* through the binding of the TF BREVIPEDICELLUS to their promoter region (Sánchez-Rodríguez et al., 2010).

Abscisic acid acts antagonistically to gibberellic acid to inhibit stem elongation by arranging longitudinally the cortical microtubules (Shibaoka, 1994). This may explain the high ABA content observed in H20, a time-point where elongation ceases (Supplementary Image S1). Genes coding for the microtubule-associated proteins, *MAP65*-8 and *MAP70-5*, were found to be highly expressed in H20. MAP65 bundles the microtubules (Soyano et al., 2008), while MAP70-5 determines the cortical patterning of the cell walls of tracheary elements (Derbyshire et al., 2015). ABA also down-regulates the expression of the FLA *AtFLA2*, whose hemp ortholog was found to be more abundant in H6 (Supplementary Table S6). AtFLA2 may induce shoot development (Johnson et al., 2003) in an auxin-dependant manner, as it forms a complex with ABCB19, a regulator of auxin eﬄux (Titapiwatanakun et al., 2009).

We were not able to significantly discriminate the bioactive cytokinin content from one time-point to another, but the lowest concentration was observed in H20 (**Figure 8C**). It has been shown that the concentration of bioactive CKs is higher in young tissues and declines as the tissue senesces (Havlová et al., 2008). However, when considering only tZ/tZR, the highest level was observed in H9 and H15 while the minimum was found in H20. It has been suggested that roots are a major site of tZ production and that tZR is the major CK present in the xylem sap (Sakakibara, 2006; Hirose et al., 2008). After translocation, tZR is metabolized to tZ, leading to signal transduction. Given the decrease of the ratios between CK nucleobases and CK nucleosides (**Figure 9**), we may suppose that there is a shift in bioactive CK allocation along the time-course. As a consequence, the fraction of bioactive CKs able to induce a signal, i.e., the nucleobases, might be higher in younger hypocotyls. At the cell wall level, CKs, and particularly tZ, are involved in the lignification of the fibers and xylem vessels (Didi et al., 2015). From our data, we may speculate that CKs regulate the initiation of lignin deposition in secondary tissues, while later lignification is tuned by other factors. *AtPRX52* (whose hemp ortholog was more expressed in H20, Supplementary Table S6) is an example of these genes regulated by several factors: it contains a high number of *Arabidopsis* response regulator 1 (ARR1)-cytokinin-binding element in its promoter region, but is also responsive to *bZIP9, bZIP50, NST1, MYB46*, or *MYB85* (Herrero et al., 2014). All these TFs were, notably, more abundant either in H15 or in H20 (Supplementary Table S4).

Methyl jasmonate and other JAs are also factors regulating the biosynthesis of cell wall building components (Pauwels et al., 2008; Men et al., 2013). This is notably the case for lignin: we have found that the expression of several methyl jasmonate-up-regulated genes peaks simultaneously with the active jasmonates content, namely orthologs of *Arabidopsis MetS1, PAL2, C3H, CCoAOMT, HCT, CAD4*, and *4CL2* (Supplementary Table S6). *IRX15-L* (glucuronoxylan backbone biosynthesis) is also up-regulated by methyl jasmonate and its expression peaked in H20.

## Conclusion

This study has highlighted the suitability of the hemp hypocotyl for the study of the main events accompanying secondary growth. Phytohormones were here shown to be tightly linked with secondary growth and cell wall deposition. Polysaccharide fingerprinting via immunohistochemistry has been linked to transcriptomic data to emphasize the role of the TFs and genes involved in the biosynthesis of the building blocks (polysaccharides, monolignols) composing the cell wall, a structure that is crucial for the development of the plant.

## Author Contributions

MB analyzed the RNA-Seq data and phytohormone profiles, performed RT-qPCR, optical microscopy and immunohi-stochemistry experiments and wrote the manuscript. SyL participated in the RNA-Seq experiment, performed the bioinformatic analysis and critically revised the manuscript. EŽ, VM, and PD performed the phytohormone analysis and critically revised the manuscript. J-FH and StL conceived the experiment and critically revised the manuscript. J-FH leads the project CANCAN. GG conceived the experiment, performed the RNA-Seq, wrote and critically revised the manuscript. All the authors have read and approved the final version of this manuscript.

## Conflict of Interest Statement

The authors declare that the research was conducted in the absence of any commercial or financial relationships that could be construed as a potential conflict of interest.

## Footnotes

1 www.predisi.de

2 http://eu.idtdna.com/calc/analyzer

## Abbreviations

CSL: cellulose synthase like
FLA: fasciclin-like arabinogalactan protein
LAC: laccase
PCW: primary cell wall
PME: pectin methylesterase
SCW: secondary cell wall
XEH: xyloglucan endohydrolases
XET: xyloglucan endotransglucosylases
XTH: xyloglucan endotransglucosylase/hydrolase
