# Supplementary figures and images for: Studying Secondary Growth and Bast Fiber Development: The Hemp Hypocotyl Peeks behind the Wall
Source: Front Plant Sci. 2016 Nov 18;7:1733. doi: 10.3389/fpls.2016.01733 (PMC5114303; doi:10.3389/fpls.2016.01733)

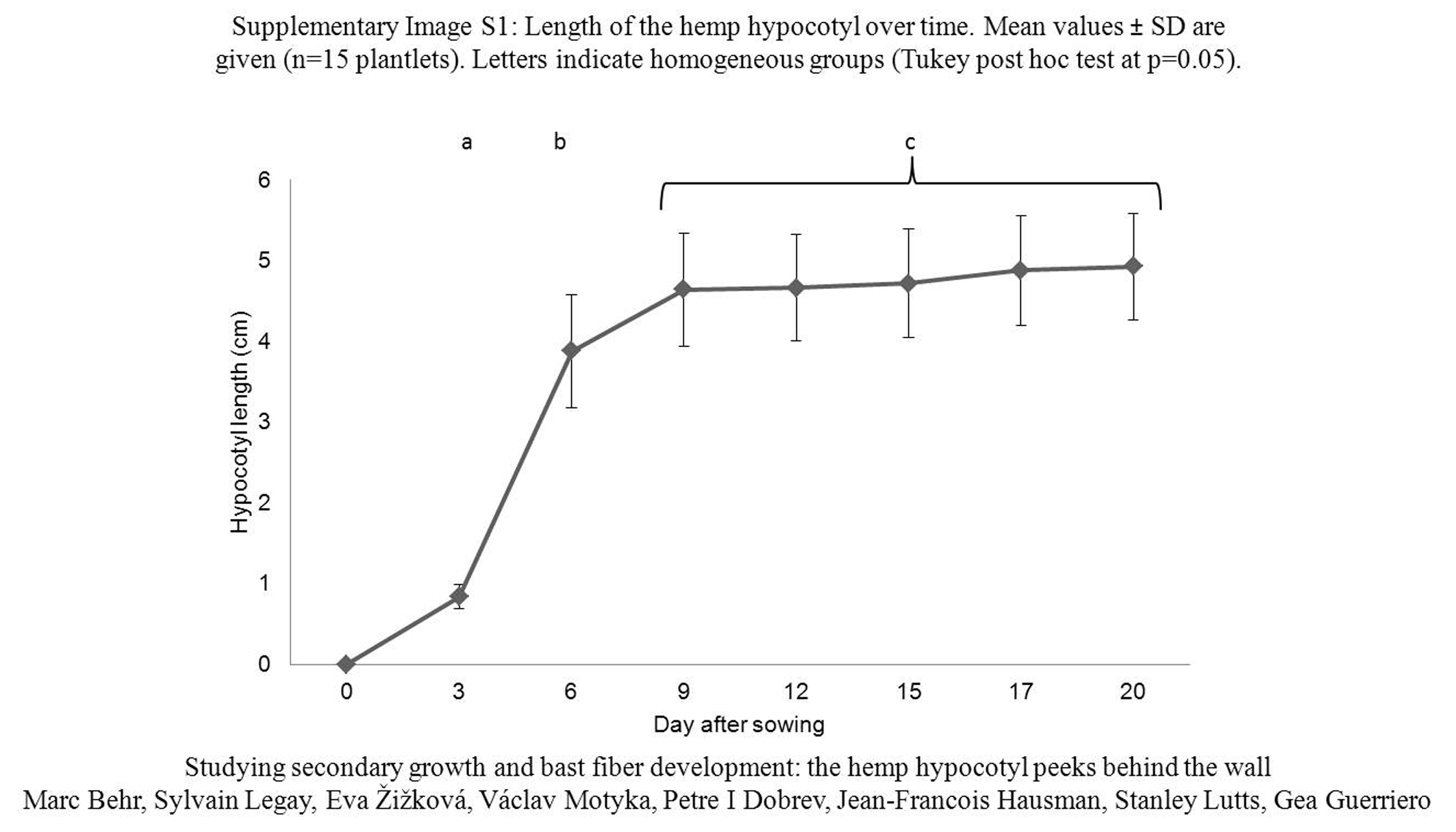

Supplement: Supplementary file 7 [file Image_1.tif]

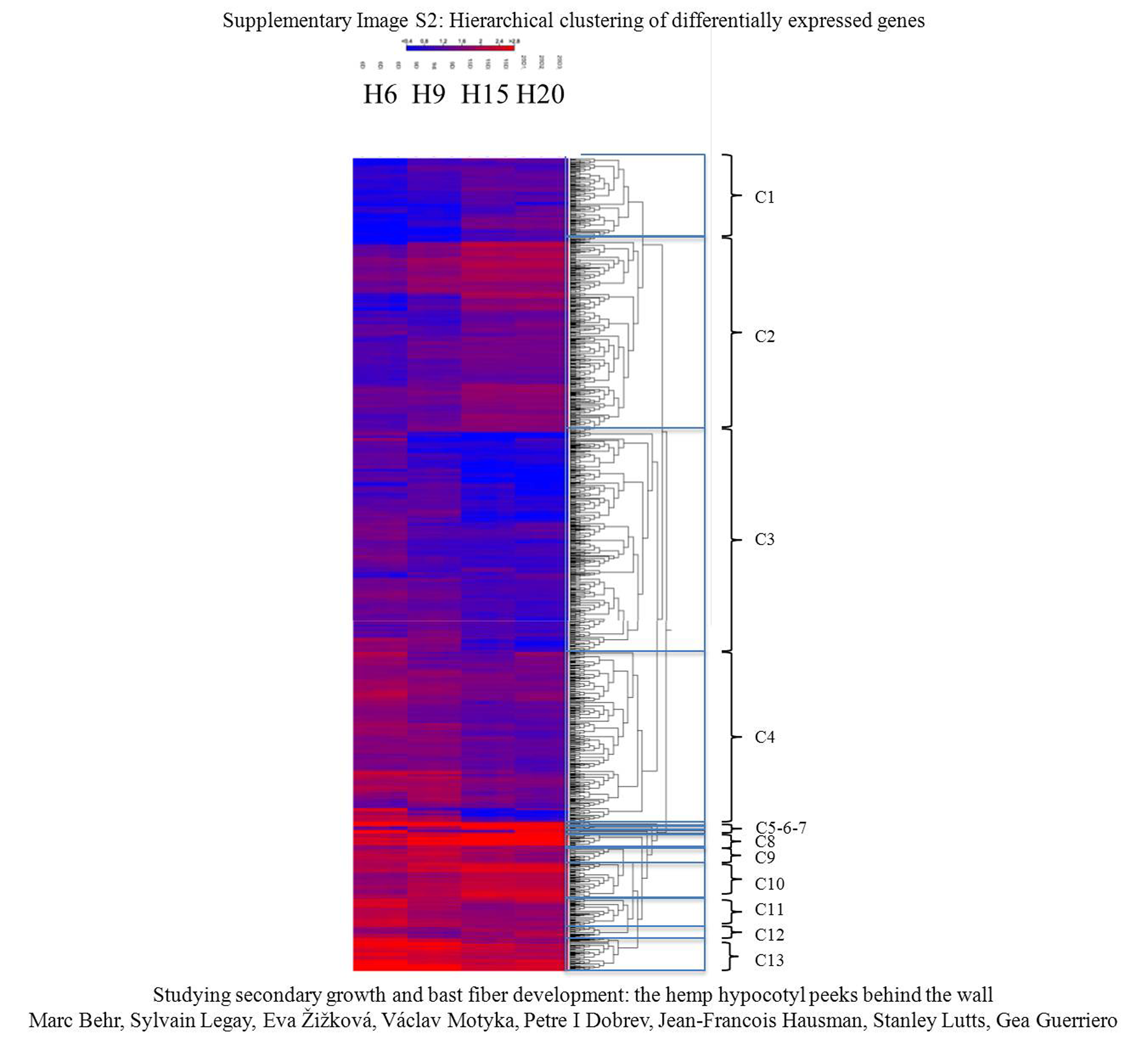

Supplement: Supplementary file 8 [file Image_2.tif]

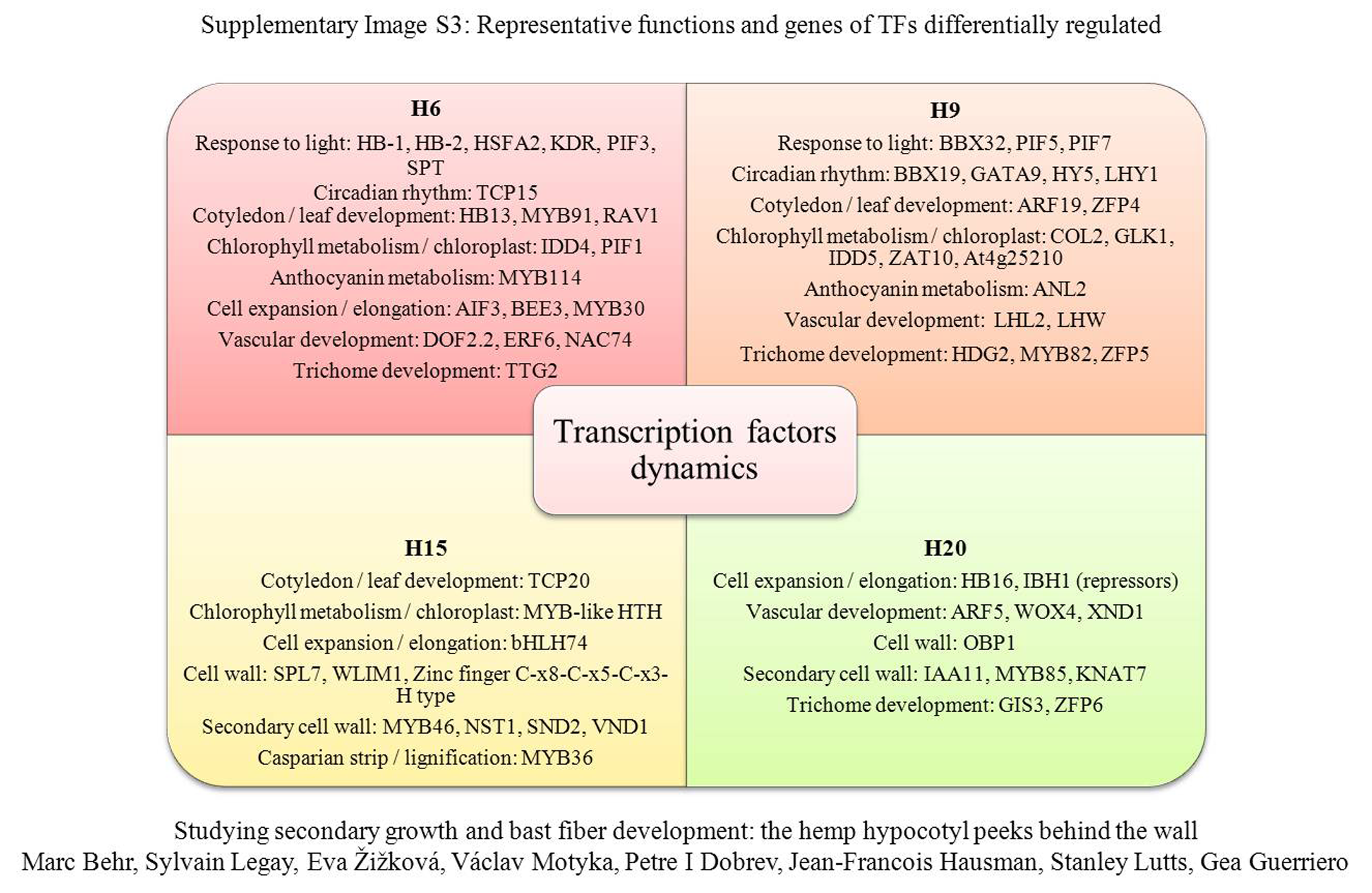

Supplement: Supplementary file 9 [file Image_3.tif]

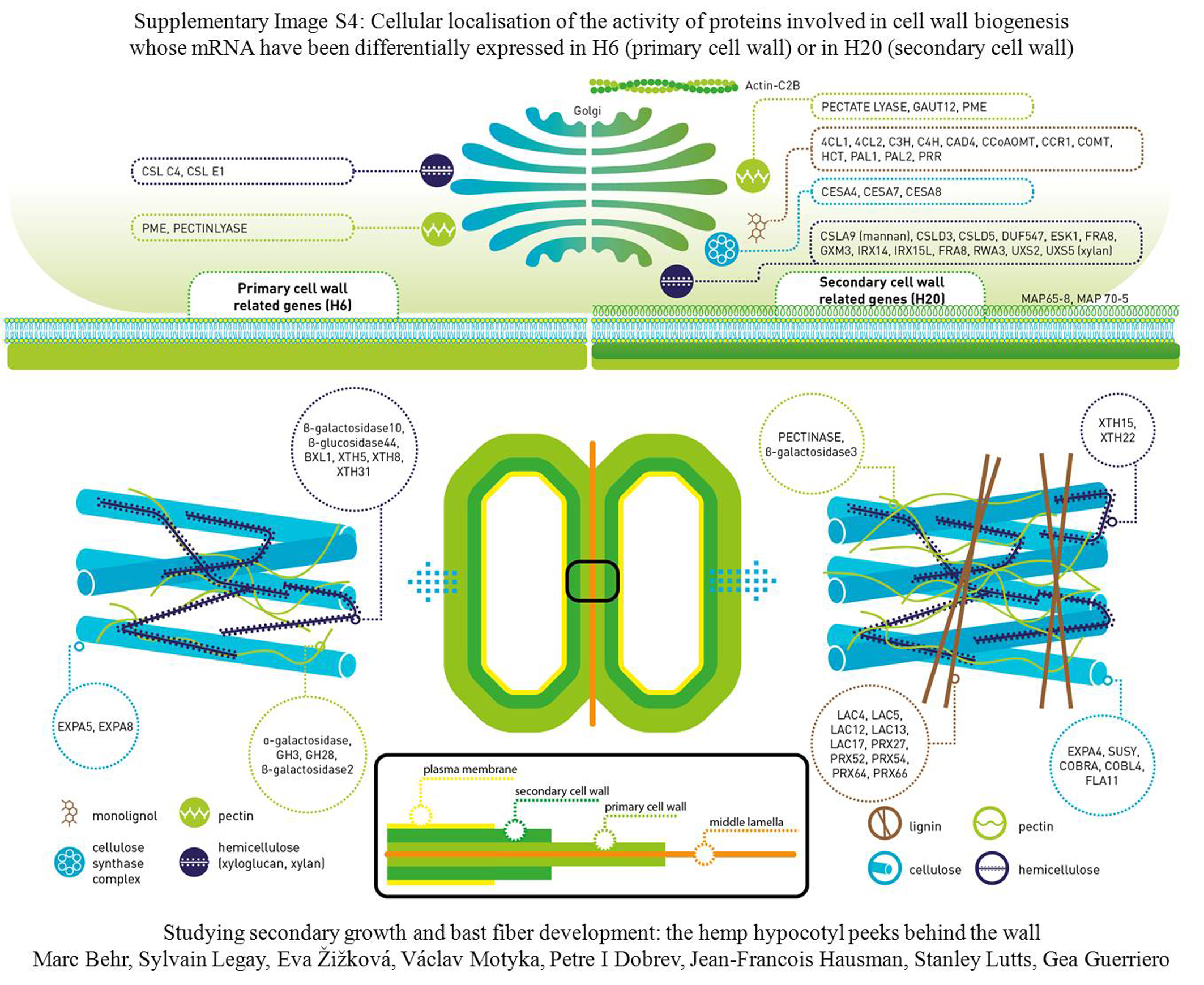

Supplement: Supplementary file 10 [file Image_4.tif]
